# Tuberculosis Joint Disease

**Published:** 1902-10

**Authors:** 


### Tuberculosis Joint Disease.

Under this head Wilson, in *New York Medical Journal,* reviews the cause and symptomatology of this often hidden disease. Laying much stress upon the appearance of muscular rigidity in the early stages of the disease, he warns against careless examination, and advises that anchylosis be the end aimed at in treatment, as striving to that end the most favorable results are often reached, while no harm in the way of acute miliary infection can be produced. C. R. M.

